# Aferindo experiências com discriminação em múltiplos grupos sociais: análise de invariância da *Escala de Discriminação Explícita* em estudantes universitários

**DOI:** 10.1590/0102-311XPT127323

**Published:** 2024-05-17

**Authors:** Fabiula Renilda Bernardo, João L. Bastos, Michael Reichenheim

**Affiliations:** 1 Universidade Federal de Santa Catarina, Florianópolis, Brasil.; 2 Faculty of Health Sciences, Simon Fraser University, Burnaby, Canada.; 3 Instituto de Medicina Social, Universidade do Estado do Rio de Janeiro, Rio de Janeiro, Brasil.

**Keywords:** Psychometrics, Bias, Surveys and Questionnaires, Social Discrimination, Psicometria, Viés, Inquéritos e Questionários, Discriminação Social, Psicometría, Sesgo, Encuestas y Cuestionarios, Discriminación Social

## Abstract

O objetivo foi avaliar a capacidade da *Escala de Discriminação Explícita* (EDE) de produzir estimativas comparáveis entre grupos de gênero, cor/raça e posição socioeconômica. A análise se baseou em dados de dois estudos, realizados com estudantes de universidades públicas brasileiras. Uma versão abreviada da EDE com oito itens foi avaliada, utilizando o método *alignment* (alinhamento). Nossos achados indicaram violação de invariância entre grupos de cor/raça e gênero. Os relatos de experiências discriminatórias tiveram melhor comparabilidade entre estratos de posição socioeconômica. Este estudo demonstrou que a EDE deve ser utilizada com cautela, especialmente para fazer comparações de estimativas de discriminação entre respondentes de cor/raça e gênero distintos. A violação de invariância observada reforça a necessidade de pesquisas adicionais, examinando se tal cenário se mantém em amostras mais amplas e diversas do país.

## Abstract (en)

This study evaluated the ability of the *Explicit Discrimination Scale* (EDS) to produce comparable estimates among respondents according to gender, color/race, and socioeconomic status. Analysis was based on data from two studies with students from Brazilian public universities. An abridged version of the EDS with eight items was evaluated by the alignment method. Findings indicated violation of invariance between color/race and gender groups. Reports of discriminatory experiences had better comparability between socioeconomic status strata. This study showed that EDS should be used with caution, especially to compare discrimination estimates between respondents of different colors/races and genders. The observed violation of invariance reinforces the need for additional research examining whether such a scenario persists in larger and more diverse samples from Brazil.

## Resumen

El objetivo fue evaluar la capacidad de la *Escala de Discriminación Explícita* (EDE) para producir estimaciones comparables entre grupos de género, color/raza y posición socioeconómica. El análisis se basó en los datos de dos estudios, realizados con estudiantes de universidades públicas brasileñas. Se evaluó una versión abreviada de la EDE con 8 ítems, utilizando el método *alignment* (alineación). Nuestros hallazgos indicaron una violación de la invariancia entre los grupos de color/raza y género. Los informes de experiencias discriminatorias fueron más comparables entre los estratos de posición socioeconómica. Este estudio demostró que la EDE debe usarse con precaución, especialmente para hacer comparaciones de estimaciones de discriminación entre encuestados distintos de color/raza y género. La violación de la invariancia observada refuerza la necesidad de investigaciones adicionales, examinando si tal escenario se mantiene muestras más amplias y diversas del país.

## Introdução

A partir dos anos 1990, observou-se um aumento expressivo no número de estudos documentando os impactos da discriminação racial e de outras formas de tratamento injusto (p.ex.: por gênero, idade, forma ou tamanho corporal) sobre a saúde física e mental ^1^^,^^2^. Apesar desse crescimento, é necessário um maior refinamento dos instrumentos utilizados na avaliação das experiências com discriminação. É preciso garantir que as escalas empregadas capturem as particularidades de diferentes segmentos populacionais e, além disso, forneçam medidas comparáveis entre si.

A *Escala de Discriminação Explícita* (EDE) ^3^ foi desenvolvida no Brasil para avaliar diferentes formas de maus-tratos, incluindo, por exemplo, aqueles motivados por raça, classe social e orientação sexual. Em sua versão original, a EDE apresenta 18 itens sobre experiências de tratamento diferencial, incluindo uma questão sobre o testemunho de discriminação perpetrada contra terceiros. A EDE avalia a discriminação percebida em três etapas e os entrevistados que relatam experiências dessa natureza são convidados a responder mais três subitens complementares acerca: (1) das possíveis motivações atribuídas ao tratamento diferencial, tais como cor ou raça, idade e classe social; (2) do grau de desconforto relacionado aos episódios; e (3) da interpretação dos eventos como discriminatórios ou não.

Desde sua elaboração inicial, a EDE vem apresentando boas propriedades configurais e métricas ^3^. Recentemente, Bastos et al. ^4^ examinaram o instrumento em estudantes universitários e propuseram uma versão abreviada, composta por oito itens, que apresentou escalabilidade aceitável. No entanto, nenhum estudo investigou se a EDE produz estimativas de discriminação comparáveis entre os grupos sociais. Investigar a capacidade de o instrumento fornecer estimativas comparáveis de discriminação em diferentes segmentos populacionais é imprescindível para a identificação válida e confiável daqueles afetados pelo problema de forma mais severa ou não ^5^. Este estudo aborda essa questão, examinando se essa versão da EDE pode estabelecer comparações entre respondentes, conforme gênero, cor/raça e posição socioeconômica.

## Métodos

Esta análise utilizou dados de dois inquéritos gerais de saúde, um realizado na Universidade Federal de Santa Catarina (UFSC) em Florianópolis (Santa Catarina), e outro, na Universidade do Estado do Rio de Janeiro (UERJ), Rio de Janeiro, Brasil. O estudo da UFSC recrutou uma amostra representativa de 1.022 alunos de graduação, por meio de um processo seletivo em dois estágios, que se estendeu de março a maio de 2012. Por sua vez, a pesquisa da UERJ foi realizada entre abril e maio de 2010 em uma amostra de 424 graduandos, selecionados por conveniência. Embora tenha sido obtida por método não probabilístico, a amostra da UERJ não foi recrutada em função de potencial interesse em responder perguntas sobre experiências com discriminação. Ambas as investigações tiveram como base questionários autopreenchíveis e foram aprovadas por seus respectivos comitês de ética em pesquisa com seres humanos (UFSC: parecer nº 459.965; UERJ: parecer nº 0016.0.259.000-08).

Os participantes foram descritos de acordo com gênero (homens e mulheres), idade, cor/raça e posição socioeconômica. Enquanto idade foi dividida em faixas etárias de 15-24, 24-34 e 35+ anos, cor/raça foi coletada segundo as categorias: branca, preta, parda, indígena e amarela. Respondentes autodeclarados amarelos (n = 15) e indígenas (n = 9) foram excluídos da análise, ao passo que pardos e pretos foram agrupados numa categoria denominada “negros”. Essa agregação é justificada por sintetizar, de um ponto de vista estatístico, a semelhança das condições socioeconômicas entre pretos e pardos no Brasil. Segundo Osorio ^6^ (p. 23), “*pretos e pardos distinguem-se bastante dos brancos, mas virtualmente diferem pouco entre si em qualquer indicador de situação ou posição social*”. Além disso, a amostra foi constituída por uma frequência pequena de autoclassificados pretos (n = 111), o que inviabilizaria uma análise separada. Por meio do Indicador Econômico Nacional ^7^, que enfatiza uma relação de bens do domicílio, os respondentes foram classificados em quintis de posição socioeconômica. Esses quintis foram subsequentemente reagrupados em duas categorias: posição socioeconômica baixa (primeiro e segundo quintis) e alta (terceiro, quarto e quinto quintis).

Nesta análise, foi considerada a versão abreviada da EDE de oito itens, a qual abrange: i1 (confundido com funcionário); i2 (tratado com desrespeito em lugares públicos); i3 (tratado com desrespeito em agências governamentais); i6 (tratado como não inteligente na escola/universidade); i10 (tratado com desprezo ao tentar namorar); i12 (tratado desrespeitosamente por parentes próximos); i13 (chamado por nomes que não gosta); e i16 (excluído por pessoas na vizinhança). As respostas aos itens foram codificadas em quatro níveis ordinais de frequência: nunca, ocasionalmente, frequentemente e sempre. Participantes que indicaram ter experiências com tratamento injusto responderam a um item adicional, questionando se foram discriminados nessas situações. Cada um dos itens foi posteriormente dicotomizado quanto à ocorrência de discriminação (sim ou não).

A capacidade de produzir estimativas de discriminação comparáveis entre grupos (i.e., invariância) foi examinada pelo método *alignment* (alinhamento) ^8^ com opção *FIXED* e parametrização *Theta*^9^, após combinação dos respondentes da UERJ e da UFSC em uma única amostra. Essa agregação se baseou em análises prévias que demonstram não haver violação de invariância nos itens da EDE em questão, particularmente quando os entrevistados são analisados conforme a instituição de ensino ^4^. Tendo em vista os itens binários, as análises empregaram modelos *probit* e o estimador *weighted least squares mean and variance* (WLSMV; mínimos quadrados ponderados ajustados pela média e variância) ^9^. O ajuste dos modelos foi avaliado por *root mean square error of approximation* (RMSEA; raiz do erro quadrático médio de aproximação), índice de ajuste comparativo de Bentler (CFI) e índice de Tucker-Lewis (TLI). RMSEA < 0,06 sugere um bom ajuste; valores > 0,10 indicam ajuste inadequado, aconselhando a rejeição do modelo ^10^. CFI e TLI com valores ≥ 0,95 indicam um ajuste aceitável ^10^. Índices de modificação também foram examinados para identificar possíveis correlações residuais entre pares específicos de itens e, caso necessário, reespecificar o modelo para obter melhor performance ^9^. A estimação de correlações residuais seguiu os critérios de melhoria do desempenho do modelo e o potencial redundância de conteúdo entre os itens em questão.

As cargas não padronizadas e os limiares dos itens foram considerados comparáveis (i.e., invariantes) quando apresentavam valor de p acima de 0,001 ^8^ no contraste entre os grupos de interesse, definidos por gênero, cor/raça e posição socioeconômica. A estatística R^2^ também foi apresentada, variando de 0 a 1, sendo valores próximos a 1 indicativos de parâmetros invariantes, enquanto valores próximos a 0, daqueles que violaram a invariância ^9^; inexiste na literatura especializada um ponto de corte específico para decidir se o parâmetro é ou não invariante a partir do R^2^. A organização do banco de dados e as análises descritivas foram conduzidas no Stata 14.1 (https://www.stata.com). Todos os testes de invariância foram executados no Mplus 8.8 (https://www.statmodel.com/), considerando os pesos e a estrutura amostral complexa na UFSC.

## Resultados

Além de terem sido excluídos os autodeclarados indígenas e amarelos, houve observações ignoradas para as classificações de gênero, idade, cor/raça e posição socioeconômica. Dessa forma, os resultados não refletem o total de entrevistados em cada instituição e variam em função da característica avaliada. Conforme observado na Tabela 1, a amostra da UERJ foi composta por 175 homens (41,4%) e 248 mulheres (58,6%), dos quais 354 (85,5%) tinham entre 15-24 anos de idade. Precisamente 244 (59,8%) entrevistados eram de posição socioeconômica alta e 164 (40,2%), de posição socioeconômica baixa. No que tange à cor/raça, 216 (52,2%) participantes se identificaram como brancos e 198 (47,8%), como negros. Na UFSC, por sua vez, 553 (54,9%) dos entrevistados eram homens e 455 (45,1%), mulheres. Tal amostra foi predominantemente constituída por indivíduos entre 15-24 anos (752; 82,2%), brancos (827; 84,5%), sendo 152 (15,5%) autodeclarados negros. Ao todo, 403 (41%) eram de posição socioeconômica baixa e 579 (59%), de posição socioeconômica alta. Quando combinadas as duas amostras, o total foi de 703 mulheres (49,1%) e 728 homens (50,9%), com a seguinte distribuição por cor/raça: 1.043 (74,9%) brancos e 350 (25,1%) negros. 823 (59,2%) entrevistados eram de posição socioeconômica alta, sendo os demais (567) de posição socioeconômica baixa.

**Tabela 1:** Distribuição dos participantes de acordo com cor/raça, gênero, posição socioeconômica, idade e instituição de ensino. Universidade do Estado do Rio de Janeiro (UERJ), Rio de Janeiro, e Universidade Federal de Santa Catarina (UFSC), Florianópolis, Brasil, 2010-2012.

Características sociodemográficas	UERJ		UFSC	
	n	%	n	%
Gênero				
Homens	175	41,4	553	54,9
Mulheres	248	58,6	455	45,1
Idade (anos)				
15-24	354	85,5	752	82,2
25-34	60	14,5	136	14,9
35+	-	-	26	0,2
Cor/Raça (autodeclarada)				
Branca	216	52,2	827	84,5
Negra	198	47,8	152	15,5
Posição socioeconômica				
Alta	244	59,8	579	59,0
Baixa	164	40,2	403	41,0

Os índices de ajuste dos modelos analisados mostraram valores de CFI e TLI ≥ 0,95; no entanto, o RMSEA apresentou valor > 0,06 para todas as classificações da amostra. Assim, o modelo que examinou grupos de cor/raça foi reespecificado segundo os índices de modificação, para a inclusão das seguintes correlações residuais: i3 e i6; i1 e i3; i6 e i16; i10 e i12. No modelo para gênero, as seguintes correlações residuais foram incluídas: i10 e i13; i6 e i16. E para a posição socioeconômica, houve inclusão de correlação residual entre i3 e i13. Após a inclusão dessas correlações, todos os modelos apresentaram valor de RMSEA < 0,06.

A Tabela 2 revela que algumas violações de invariância foram identificadas. Entre brancos e negros, três itens foram considerados não invariantes quanto às cargas: i2, i3 e i13. Identificaram-se, também, dois itens com limiares não invariantes: i1 e i10. Nos grupos de gênero, outras violações foram detectadas: a carga e o limiar do item i6; ao passo que os itens i2 e i12, somente de limiar. Houve violação de invariância segundo posição socioeconômica baixa e alta no limiar do i1 e na carga do i16. Ao inspecionar a estatística de R^2^, observou-se que alguns itens considerados invariantes entre os grupos de gênero e posição socioeconômica apresentaram valor zero para cargas e limiares.

**Tabela 2:** Cargas não padronizadas e limiares da *Escala de Discriminação Explícita* (versão reduzida, de oito itens), de acordo com cor/raça, gênero e posição socioeconômica. Universidade do Estado do Rio de Janeiro (UERJ), Rio de Janeiro, e Universidade Federal de Santa Catarina (UFSC), Florianópolis, Brasil, 2010-2012.

Item	Cargas fatoriais não padronizadas				Limiares			
Cor/raça	Brancos	Negros *	Valor de p	R^2^	Brancos	Negros	Valor de p	R^2^
i1	0,888	0,761	0,361	0,677	3,001	1,434	0,000	-
i2	0,825	1,555	0,000	-	0,886	0,706	0,274	0,750
i3	0,621	0,910	0,000	-	1,800	1,875	0,496	0,877
i6	0,841	0,808	0,389	0,935	0,948	0,964	0,624	0,985
i10	0,827	0,801	0,512	0,954	1,504	1,179	0,000	-
i12	0,925	0,976	0,463	0,308	1,264	1,287	0,525	0,992
i13	1,045	1,570	0,000	-	0,487	0,629	0,071	0,819
i16	0,984	0,910	0,349	0,841	2,046	1,613	0,034	0,548
Gênero	Homens	Mulheres **	Valor de p	R^2^	Homens	Mulheres	Valor de p	R^2^
i1	0,845	0,973	0,460	0,000	2,148	2,093	0,475	0,924
i2	1,032	1,073	0,515	0,000	0,984	0,671	0,000	-
i3	0,796	0,700	0,384	0,554	1,735	1,916	0,027	0,000
i6	1,166	0,640	0,000	-	1,405	0,750	0,000	-
i10	0,986	0,780	0,201	0,385	1,336	1,481	0,015	0,000
i12	1,020	0,752	0,039	0,320	1,450	1,122	0,000	-
i13	0,959	1,225	0,124	0,000	0,512	0,516	0,921	0,992
i16	0,805	1,004	0,226	0,000	1,683	1,926	0,026	0,000
Posição socioeconômica	Alta ***	Baixa	Valor de p	R^2^	Alta	Baixa	Valor de p	R^2^
i1	0,798	0,598	0,233	0,000	1,958	1,514	0,000	-
i2	0,816	0,990	0,211	0,230	0,423	0,546	0,073	0,000
i3	0,946	0,747	0,159	0,000	1,712	1,704	0,897	0,984
i6	0,678	0,881	0,006	0,178	0,783	0,646	0,027	0,910
i10	0,840	0,839	0,962	0,992	1,163	1,197	0,553	0,977
i12	0,805	0,962	0,190	0,245	0,997	1,077	0,239	0,627
i13	1,234	1,225	0,872	0,854	0,210	0,160	0,119	0,986
i16	0,986	0,754	0,001	0,000	1,563	1,561	0,972	0,985

* Modelo reespecificado para incluir correlações residuais entre i3 e i6, i1 e i3, i6 e i16, i10 e i12;

** Modelo reespecificado para incluir correlações residuais entre i10 e i13, i6 e i16;

*** Modelo reespecificado para incluir correlação residual entre i3 e i13.

## Discussão

A EDE é utilizada para mensurar diversas formas de discriminação e seus impactos na saúde. Alguns estudos ^3^^,^^4^^,^^11^ já demonstraram que o instrumento apresenta boas propriedades configurais, métricas e escalares, mas o pressuposto de que a escala produz estimativas comparáveis entre diferentes grupos de respondentes permaneceu, até agora, inexplorado. Isso é de extrema importância, pois estimativas de discriminação obtidas em variados segmentos populacionais não podem ser contrastadas com validade e confiabilidade se a invariância do instrumento não tiver sido previamente demonstrada.

Nosso estudo preencheu parcialmente essa lacuna de conhecimento, avaliando a invariância da EDE entre grupos de cor/raça, gênero e posição socioeconômica em uma amostra de graduandos. Os achados revelaram que cinco parâmetros violaram a invariância (cargas não padronizadas: i2, i3 e i13; limiares: i1 e i10) entre os grupos raciais, sendo essa quantidade de itens com violação substancialmente maior do que o recomendando ^8^. Também observamos que as medidas de discriminação carecem de invariância entre os grupos de gênero, com identificação de quatro parâmetros violantes (uma carga não padronizada: i6; e três limiares: i2, i6 e i12). No que tange aos grupos baseados na posição socioeconômica, o pressuposto de invariância também não foi atendido, sendo identificados dois parâmetros (limiar do i1 e carga do i16) com violação entre os grupos.

Os resultados se somam aos de pesquisa anterior ^12^, que havia sinalizado outros problemas importantes na EDE entre estudantes universitários. Cabe pontuar, ainda, o fato de alguns itens terem apresentado R^2^ com valor zero para cargas e limiares. Tal observação pode estar relacionada ao pequeno número de grupos ou ao tamanho reduzido da amostra, os quais podem implicar problemas com os cálculos de estatística em questão ^9^. É importante considerar que a amostra utilizada para avaliar o instrumento é formada por graduandos expressivamente jovens, os quais representam um segmento pequeno e específico da população brasileira. Dessa forma, suas percepções sobre eventos discriminatórios podem ser marcadamente diferentes de outros grupos populacionais.

Apesar dessas particularidades, os achados reforçam a necessidade de pesquisas que examinem se esse cenário se mantém em outras populações do país. Além da invariância, futuras pesquisas sobre as propriedades psicométricas da EDE devem analisar as múltiplas correlações residuais observadas. Examinar, por meio de abordagens qualitativas e quantitativas, se essas correlações refletem alguma redundância de conteúdo ou idiossincrasias da amostra investigada contribuirá para um refinamento do instrumento ou sua manutenção na forma atual. Outra questão que deve ser enfrentada é a invariância da EDE entre pardos e pretos, além de segmentos populacionais atravessados por múltiplos eixos de marginalização. Espera-se que a avaliação e o refinamento contínuos da EDE contribuam para o fortalecimento de pesquisas que estabelecem vínculos entre injustiça social, condições e iniquidades em saúde.
